# Physics-informed neural networks for high-resolution weather reconstruction from sparse weather stations

**DOI:** 10.12688/openreseurope.17388.1

**Published:** 2024-05-10

**Authors:** Álvaro Moreno Soto, Alejandro Cervantes, Manuel Soler

**Affiliations:** 1 Department of Aerospace Engineering, Universidad Carlos III de Madrid, Leganés, Community of Madrid, 28911, Spain; 2 Escuela Superior de Ingeniería y Tecnología, Universidad Internacional de La Rioja, Logroño, La Rioja, 26006, Spain

**Keywords:** Artificial intelligence, machine learning, physics-informed neural networks, weather reconstruction, fluid mechanics, fluid flows

## Abstract

**Background:**

The accurate provision of weather information holds immense significance to many disciplines. One example corresponds to the field of air traffic management, in which one basis for weather detection is set upon recordings from sparse weather stations on ground. The scarcity of data and their lack of precision poses significant challenges to achieve a detailed description of the atmosphere state at a certain moment in time.

**Methods:**

In this article, we foster the use of physics-informed neural networks (PINNs), a type of machine learning (ML) architecture which embeds mathematically accurate physics models, to generate high-quality weather information subject to the regularization provided by the Navier-Stokes equations.

**Results:**

The application of PINNs is oriented to the reconstruction of dense and precise wind and pressure fields in areas where only a few local measurements provided by weather stations are available. Our model does not only disclose and regularize such data, which are potentially corrupted by noise, but is also able to precisely compute wind and pressure in target areas.

**Conclusions:**

The effect of time and spatial resolution over the capability of the PINN to accurately reconstruct fluid phenomena is thoroughly discussed through a parametric study, concluding that a proper tuning of the neural network’s loss function during training is of utmost importance.

## Plain language summary

There is currently a great interest in the many uses of artificial intelligence (AI) and how it is affecting our daily lives. From the robotics field to the use of language recognition to interact with different users, we are experiencing how machine intelligence is increasing day by day. In this article, we delve into one of the many applications of artificial intelligence: weather reconstruction. The ability to accurately determine weather conditions is believed to have an impact on various disciplines, e.g. reducing costs at airports due to delays, cancellations and associated compensations. In this particular example, a precise description of the status of the atmosphere is therefore necessary if countermeasures are to be executed. However, conventional weather recording with on-ground stations is often limited to a few sparse locations. Following that line of thought, it is not only necessary to estimate the weather in areas surrounding stations, but also on other target areas which may be subject to lack of weather information. Our strategy is based on the application of neural networks, a type of AI architecture, to infer data based on the underlying physics that drive the measured weather phenomena. For that purpose, we make use of neural networks which are consistent with physics laws, the so-called physics-informed neural networks (PINNs). This article deals with their adoption to weather pattern reconstruction, with the objective of further increasing the precision and availability of information given scarce reference measurements.

## 1. Introduction

Extreme weather conditions have a major impact, not only on nature, but also on human-based operations. Weather-related disasters have been reported to incur in substantial costs, exceeding USD 92.9 billion in the United States of America in 2023 ^1^ and EUR 52.3 billion in the European Union in 2022 ^2^, adding up to USD 2.655 trillion and EUR 650 billion since 1980, correspondingly. Here, not only the effect of storms is accounted for, but also extreme temperatures, floods and other natural catastrophes ^3^. The ability and capacity to properly reconstruct, predict and prevent weather disasters are therefore of crucial importance, since not only human lives can be saved, but the associated costs may be remarkably decreased. The art of weather forecasting, although ancient, spans throughout history (the interested reader is referred to a documentation on Babylonians using haloes and appearances of clouds for storm prediction ^4^; Aristotle’s treatise *Meteorologica* – see e.g. its English translation ^5^ – on the formation of rain, clouds, hail, wind, thunder, lightning, and hurricanes; and more recently the theory of chaos proposed by Lorenz and its application to ‘Deterministic nonperiodic flow’ ^6^, which was to revolutionize the statistical treatment of weather data). However, since the beginning of the computer age and with an increasing number of weather stations (WS) around the world, dedicated satellites orbiting the Earth and high precision weather gauges, weather reconstruction and now-/fore-casting has become a more reliable science thanks to the boost of numerical methods ^7^.

Nowadays, access to massive amounts of data has leveraged the use of neural networks (NNs), one of the many applications of artificial intelligence, in many fields in which data assimilation becomes a cornerstone. The capacity of NNs to find intrinsic correlations with complex data has widened the possibilities of application in fields where time sensitivity and computational effort may not work in favor. Novel studies have promoted the analysis of weather information using convolutional neural networks (CNNs) and the associated recommendations to undertake when storms are approaching critical areas, such as the vicinity of airports ^8, 9^. Recently, these architectures have gained substantial precision and adaptability due to the development of autoencoders and Fourier neural operators, which have demonstrated excellent performance when working with scarce noisy information ^10, 11^. Other architectures, such as recurrent neural networks (RNNs), in particular long short-term memory (LSTM), have been shown to provide precise forecasting of weather phenomena given sequences of measurements at a set of fixed locations ^12^. However, even though these NNs have shown excellent performance when working with extensive sets of structured data, they lack a regulatory basis upon which to extract conclusions based on a physically viable behavior.

Since the development of physics-informed neural networks (PINNs), a type of NN which incorporates physics constraints during its training stage ^13, 14^, their versatility has promoted their application in the vast field of fluid mechanics, as the embedding of the well-known Navier-Stokes (NS) equations can be achieved and becomes a source of regularization when data-assimilating ^15– 18^. The compliance with this system of equations becomes a relevant asset for the reconstruction of weather events ^10^, more particularly the wind direction and the pressure of the atmosphere at a certain moment in time. Even though PINNs are not fully capable of solving a complete formulation of the NS equations given their extreme complexity, they can outperform direct numerical simulations (DNSs) when solving Reynolds-averaged NS equations as far as their precision and, most importantly, their computational cost are concerned ^19^. PINNs are able to go beyond the limitations of experimental procedures, since they can account for behavior which cannot be measured directly by current technology ^14, 20^ or due to scarcity of resources, e.g. limited amount of probes in a water / wind tunnel experimental setup ^21, 22^. In addition, reference information needs not be structured on a regular grid, which is a major advantage as compared to other architectures. Current trends in the improvement of PINN architectures are heading towards the development of physics-informed neural operators which intrinsically embed the physics constraints without the need of investing in automatic differentiation for the computation of derivatives ^23^. Still, PINNs have shown to be extremely dependent on the time and spatial resolution of the available data. Recent research lines showcase alternative methods to enhance time resolution provided that spatial resolution and a set of time-resolved pointwise measurements are at hand ^22^. Nonetheless, PINNs have been demonstrated to perform in an excellent manner with datasets originated in controlled environments, such as numerical simulations or lab experiments. However, there is still room for improvement and the use of PINNs on real data lacking both time and spatial resolution has not been yet tested to the best of the authors’ knowledge. This scenario is very representative of weather reconstruction, in which from a limited WS set, the full fluid field needs to be inferred. In this case, information from sparse networks of sensors can be leveraged to provide fast and accurate predictions. This is essential for many fields, among them the organization of air traffic, both in the air and ground stage, especially in areas surrounding or approaching airports’ vicinities ^24– 26^.

This article deals for the first time with the reconstruction capacity of a standard PINN given the information provided by a sparse WS set, whose recordings may not be exactly accurate and incorporate experimental noise. The tuning of the PINN to best adapt to the reference information while in parallel imposing a regularization based on a physics constraint is exposed here, showing that the precision of the final reconstruction is highly dependent on the quality of the reference information and the way in which the different contributions to the loss function are weighted. The article is organized in the following manner: Section 2 introduces the PINN architecture and the necessary pretreatment of the available data to guarantee a proper performance of the NN; Section 3 discusses the accuracy of the weather reconstruction and the different effects that time and spatial resolution have on the final output of the PINN. Additional counteractive strategies are proposed to compensate for the lack of both resolutions in the original dataset; finally, Section 4 discloses the conclusions of the article.

## 2. Methods

The proposed architecture is based on a standard PINN as described in the original article by 13: a fully connected NN in which each neuron of each hidden layer is connected to all the neurons of the subsequent one. PINNs are capable of embedding constraints based on physics laws to regularize and supervise the training of the NN in such a way that this process is considered semi-supervised: the NN learns both from labeled and unlabeled data, being the labeled data the information provided by WS and the unlabeled data the physics constraints. The ability to adapt physics laws is achieved via automatic differentiation during backward propagation ^27^. Here, the derivatives of the outputs versus the inputs can be calculated with precision and allow for the computation of residuals from ordinary or partial differential equations ^28^. For the case of fluid flow characteristics, such as wind and pressure profiles, the Navier-Stokes (NS) equations can be assumed to drive the evolution of the weather phenomena. In a simplified manner, NS equations may be written as follows:


$$\begin{array}{l} ■\;\frac{\partial u}{\partial x}+\frac{\partial v}{\partial y}+\frac{\partial w}{\partial z}=0\;(e_{1})\\ ■\;\frac{\partial u}{\partial t}+\left(u\frac{\partial u}{\partial x}+v\frac{\partial u}{\partial y}+w\frac{\partial u}{\partial z}\right)+\frac{\partial p}{\partial x}-\frac{1}{Re}\left(\frac{\partial^{2}u}{\partial x^{2}}+\frac{\partial^{2}u}{\partial y^{2}}+\frac{\partial^{2}u}{\partial z^{2}}\right)=0\;(e_{2})\\ ■\;\frac{\partial v}{\partial t}+\left(u\frac{\partial v}{\partial x}+v\frac{\partial v}{\partial y}+w\frac{\partial v}{\partial z}\right)+\frac{\partial p}{\partial y}-\frac{1}{Re}\left(\frac{\partial^{2}v}{\partial x^{2}}+\frac{\partial^{2}v}{\partial y^{2}}+\frac{\partial^{2}v}{\partial z^{2}}\right)=0\;(e_{3})\\ ■\;\frac{\partial w}{\partial t}+\left(u\frac{\partial w}{\partial x}+v\frac{\partial w}{\partial y}+w\frac{\partial w}{\partial z}\right)+\frac{\partial p}{\partial z}-\frac{1}{Re}\left(\frac{\partial^{2}w}{\partial x^{2}}+\frac{\partial^{2}w}{\partial y^{2}}+\frac{\partial^{2}w}{\partial z^{2}}\right)=0\;(e_{4})\;(1) \end{array}$$

Here, *t*, *x*, *y*, *z* are dimensionless time and spatial coordinates in a Cartesian system of reference, and *u*, *v*, *w* are dimensionless velocity components in their corresponding spatial direction, whereas *p* refers to the dimensionless pressure. *Re* defines the dimensionless Reynolds number. The terms *e _i_* ( *i* = 1,…,4) within parenthesis indicate the residuals of the equations, which will be explained later in the section. For applications to weather reconstruction at ground level, winds in the vertical direction, though essential for vertical wind shear, typically represent one or two orders of magnitude smaller than horizontal wind speeds ^29– 31^. Therefore, the term *w* may be safely neglected. In addition, WS seldom provide information in the vertical direction and generically measure in a horizontal plane. Therefore, in case vertical fluctuations are not available, all terms with respect to the *z* coordinate need be eliminated. This introduces a loss of accuracy and limits the implementation of the methodology to cases that are 2D on average.

Consequently, the PINN receives as input the two spatial coordinates *x*, *y* and time *t*, and extracts the in-plane velocity components *u*, *v* and pressure *p*. Our PINN is based on a fully connected NN composed of *N _l_* = 12 hidden layers (among which the first 8 are activated by a hyperbolic tangent function, leaving the rest with a linear activation) with number of neurons *N _n_* = 600. The loss function to be minimized during training combines two main contributions:

- the error with respect to the reported data from WS, ℒ _WS_,
- and the residuals obtained from the NS equations, ℒ _NS_.

In detail, *L*_WS_ = *ω _u_ ℒ _u_* + *ω _v_ ℒ _v_* + *ω _p_ ℒ _p_*, being ω_*_ = ℒ _*_/( ℒ _NS_ + *ℒ _u_* + *ℒ _v_* + ℒ *_p_*), where the subindex represented by the bullet • refers to the corresponding variable measured by WS. The associated loss contribution of each component is calculated based on the mean squared error (MSE) with respect to the reference data, computed in the following form:


$$\text{MSE}(\tilde{Z},Z)=\frac{1}{N_{t}N_{\text{WS}}}\sum_{i=i}^{N_{t}}\sum_{j=1}^{N_{\text{WS}}}{\left(\tilde{Z}(T_{i},X_{j})-Z(T_{i},X_{j})\right)}^{2}\;(2)$$

where *_i_* and *_j_* indicate discrete time and position instances over *N _t_* number of times for *N*_WS_ number of weather stations, and $\tilde{Z}$ and correspond to the predicted and real values of a particular variable, respectively. For dimensionality purposes, the MSE will be referred to the squared standard deviation value of each variable, so as to account for the overall corresponding fluctuations within the full time and spatial frame during the learning process. Consequently, ℒ** = MSE( $\tilde{Z}$ , )/( *σ*( )) ^2^, where *σ* indicates the standard deviation. It is important to note that, given the uncertainty of experimental measurements, real values may not be by default accurate, which exposes a limitation of the PINN when working with noisy information.

On the other hand, for the case of a 2D fluid flow, the physics-constraint based on Equation 1 reduces to three equations, i.e. continuity and two momentum equations, resulting in ℒ _WS_ = MSE( *e*_1_,0) + MSE( *e*_2_,0) + MSE( *e*_3_,0), where 0 would be the reference value for a perfectly compliant fluid domain and *e _i_* ( *i* = 1,2,3) correspond to the residuals already introduced in Equation 1. Such residuals are calculated by substituting the predicted values from the PINN and their corresponding derivatives via automatic differentiation into the defined equations. It must be remarked that, whereas the MSE definition used in ℒ _WS_ only covers the points in space with a WS location, the corresponding MSE used for ℒ _NS_ spans to the entire field of flow reconstruction, i.e. the term *N*_WS_ in Equation 2 needs to be substituted by *N _S_* ≫ *N*_WS_, where *N _s_* refers to the number of spatial points in the field of reconstruction.

The final loss may be then expressed as a sum of the two independent losses. However, we opt for the adaptive weighted alternative introduced in 22, namely:


$$ℒ=\omega_{\text{NS}}{ℒ}_{\text{NS}}+{ℒ}_{\text{WS}},\;(3)$$

where *ω*_NS_ is computed analogously to the previous weights. Therefore, the total loss may be rewritten as ℒ = $\left({ℒ}_{NS}^{2}+{ℒ}_{u}^{2}+{ℒ}_{v}^{2}+{ℒ}_{p}^{2}\right)$ /( ℒ _NS_ + ℒ *_u_* + ℒ *_v_* + ℒ *_p_*). This weighted definition of the loss function works as an adaptive compensation between two counteractive events ^22^: the homogenizing effect caused by the imposition of the NS equations which tends to smooth derivatives to achieve a null residual (indeed, an homogeneous velocity and pressure fields perfectly comply with NS equations), and the observance of reported data at the locations where WS are positioned.

Finally, by inspecting Equation 1, the only field strictly necessary for regularization would be the wind velocity, since the reference pressure values are used solely for the purpose of recovering the pressure field. Indeed, the PINN supplies the pressure gradient, thus any pressure measurement at any arbitrary point in the domain would suffice for a comprehensive reconstruction. Whereas the continuity equation inherently regularizes the velocity field, the inclusion of the momentum equations further enhances regularization by revealing the pressure gradient. It is important to note that setting a suitable pressure boundary condition in at least one point is crucial. As discussed in 22, the pressure gradient in the loss function acts as a ‘residual drain’ and incorporates errors from inaccuracies in other terms of the equations or missing terms (e.g. the out-of-plane motion expanding to the vertical direction). The inclusion of numerous pressure reference values improves convergence and enhances flow reconstruction, since they impede the accumulation of residuals in the pressure gradient, thereby augmenting the regularization of the velocity fields.

### 2.1. Data pretreatment prior to NN training

Generically, information provided by WS originates from ASOS (automated surface observing system) or METAR (meteorological aerodrome reports). Typically, each WS is specifically targeted to register a set of variables, and their quality and recording frequency may fluctuate. Particularly for this article, the dataset contains the following information: date, longitude, latitude, altitude, temperature, wind speed, wind direction and pressure. However, data preprocessing is of utmost importance since the training information needs to be clean of corrupted or missing information. First of all, information from the *date* field needs to be transformed into continuous time measurements in seconds. For that purpose, the use of Python *datetime* library is recommended. As a result, time information *T* may be obtained. Secondly, the location of each weather station needs be transformed into Cartesian coordinates. Spherical projections are applied to longitude and latitude so that information can be retrieved on a 2D plane. Consequently, original values given in degrees for latitude and longitude may be translated to meters in Cartesian horizontal and vertical coordinates *X* and *Y*, respectively. For the altitude component *Z*, no transformation is needed as such information is already expressed in meters over sea level (SL). A similar situation occurs with temperature data *C*, given by default in Celsius. Regarding the velocity field, wind direction is normally given in degrees with respect to the North, in which the positive angle moves clockwise. Therefore, given the information of wind speed and wind direction, the two velocity components *U* and *V* may be easily recovered on the Cartesian coordinates *X* and *Y*. Pressure *P* is generically given in mbar, so an appropriate multiplication by 100 transforms those values into Pa. In addition, *P* may be transformed to its equivalent condition at a specific altitude, e.g. sea level (SL). This conversion may reduce the error committed by the assumption of 2D flow from a 3D domain. Pressure has a strong dependence on altitude and temperature. Following the international standard atmosphere (ISA), the equivalent pressure value at SL at standard temperature conditions (15°C) can be calculated according to the following equation:


$$P_{\text{SL}}=P{\left(1-\frac{0.0065Z}{C+273.15+0.0065Z}\right)}^{-5.257},\;(4)$$

where *P*_SL_ is the equivalent pressure at SL for the associated measured *P* at any other altitude *Z* and temperature *C*. Once all relevant variables have been translated to their corresponding 2D simplification in SI units, it is extremely convenient to transform them to dimensionless variables, both to facilitate the interpretation of the order of magnitude of the available information and simplify the training process of the PINN, since Equation 1 can be thus directly computed. Reference distance *L*, velocity *W* and pressure *P*_0_ are to be defined, namely:


$$\begin{array}{l} ■\;L=\sqrt{{\left(X_{\text{max}}-X_{\text{min}}\right)}^{2}+{\left(Y_{\text{max}}-Y_{\text{min}}\right)}^{2}}\\ ■\;W=\sqrt{|U{|}_{\max}^{2}+|V{|}_{\max}^{2}}\\ ■\;P_{0}=\bar{P},\;(5) \end{array}$$

where subindexes *max* and *min* refer to the maximum and minimum values of the corresponding variable, absolute values are indicated between bars and the overhead bar represents the mean value. Consequently, location, time, velocity and pressure may be non-dimensionalized as follows:


$$\begin{array}{l} ■\;x=X/L\;■\;y=Y/L\;■\;t=TW/L\\ ■\;u=U/W\;■\;v=V/L\;■\;p=(P_{\text{SL}}-P_{0})/(\rho W^{2})\;(6) \end{array}$$

These dimensionless variables can be directly substituted in Equation 1, in which *Re* is defined as *Re* = *ρWL*/ *μ*, being *ρ* the air density and *μ* its dynamic viscosity (for simplification purposes, both properties are referred to the standard temperature).

Finally, it is typical that, within a dataset involving WS measurements, empty records may occur. As PINNs should be trained on complete records of data, either incomplete information (usually indicated as Not a Number - NaN) need be removed or data attribution techniques must be used to estimate the missing values. In this article, we opt for the first option and NaN values are removed from the training dataset.

## 3. Experiments and results

**Figure 1.**
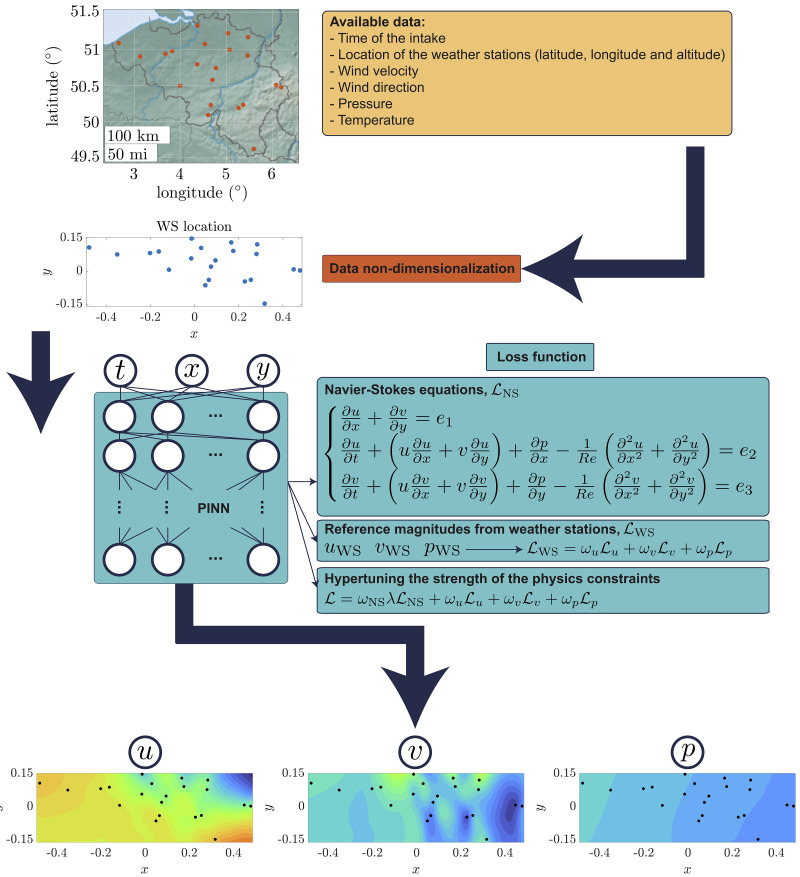
Sketch of the proposed methodology discussed in Section 3. First, a real dataset containing weather information provided by a fix number of WS is used to train a PINN after a process of data non-dimensionalization (refer to Subsection 2.1 for a detailed description). The PINN considers two main contributions during its learning process: the residuals from the NS equations and the error with respect to experimentally accessible data. The tuning of the strength of the physics constraints, thoroughly explained in Subsection 3.2, is of crucial importance when reference information is scarce and potentially subject to noise. The final output of the process is a detailed fluid field disclosing both wind components in horizontal and vertical directions and pressure.

This section discusses the application of the methodology explained before to a dataset consisting of measurements from 21 WS in the region of Brussels-Zaventem airport for a period of 14 days in the year 2018. In general, data sources indicate wind velocities and pressure values on a scattered 3D distribution, since each WS has a determined location on the horizontal plane given by its latitude and longitude and a different altitude over sea level (SL). However, the standard deviation of the latter for this particular dataset has a value of 180 m, which according to 32 falls within the assumption of low wind profiles, which states that wind velocities could be considered quasi-constant over a distance below 500 m over ground level, ignoring the contribution of the first few meters (the so-called wind boundary layer). On this basis, a 3D domain may be simplified to a 2D domain, exposing a limitation of the PINN to accurately reconstruct 3D information given a very scarce set of reference information. Our weather reconstruction is therefore presented in a 2D format, for which every parameter depending on altitude has been transformed to their sea-level equivalent according to the international standard atmosphere (ISA), as already detailed in the previous Subsection 2.1. A description of the methodology is outlined in Figure 1.

Three experimental settings using the same data are proposed, each of them with a different objective. First, the standard architecture is designed to reconstruct the true field using an output grid of varying spatial resolution. This experiment assesses the impact of the resolution parameter *R* on the final reconstruction capacity. Second, the effect of the hyperparameter tuning the strength of the physics constraint is measured. Third, a series of validation experiments are performed to determine the generalization capabilities of the PINN on unseen scenarios.

### 3.1. Assessing the effect of temporal and spatial resolution

The reconstruction ability of the PINN highly depends on the spatial and time resolution of the original dataset used during training. For this particular case, the minimum time resolution corresponds to 10 min, which is the time interval between each intake of the WS. Considering that our field of view covers a reference length *L* ≈ 410000 m with a reference velocity *W* ≈ 17 m/s, the time interval to cross the full domain is *τ* = *L*/ *W* ≈ 82000 s ≈ 1367 min. Therefore, the available time resolution should be enough to capture the majority of events occurring in the domain of observation. However, faster-developing phenomena under time resolution may not be recovered. In addition, the errors committed by the temporal derivatives in Equation 1 tend to homogenize the final reconstruction and again pose a limitation of the methodology ^22^. A potential technique to further improve the given resolution may involve the parallel recording with high-frequency probes, as explained in 22. In addition, viscosity effects in Equation 1 may be neglected, since for a typical kinematic viscosity at standard conditions (temperature of 15°C, pressure of 1 atm) *v* = 1.46 × 10 ^–5^ m ^2^/s, *Re* = *LW*/ *v* = 4.774 × 10 ^11^. This adds an additional layer of simplification, which accelerates the convergence of the PINN to the detriment of recovering potential viscosity-induced events.

**Figure 2.**
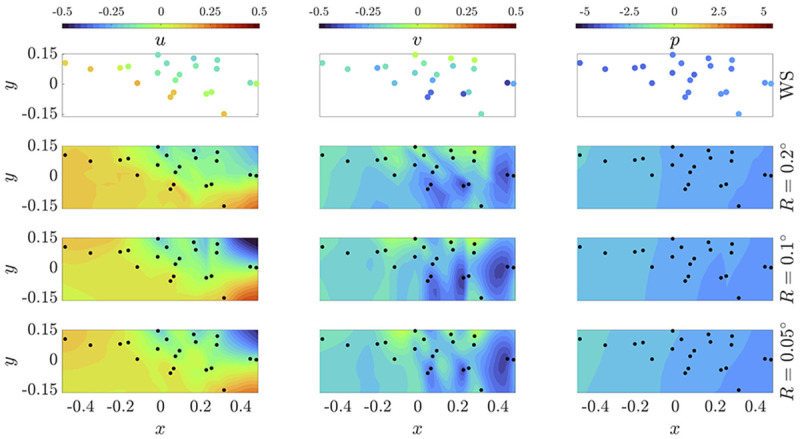
Reconstruction of wind velocity and pressure fields from measurements given by a WS set via PINN. The outcomes in each row correspond to a different spatial resolution *R*. The reference data for this specific time intake is shown in the first row as guideline. A video entitled *PINN_resolution.mp4* disclosing the weather evolution for 14 days of recording is submitted along this article.

Concerning spatial resolution, PINNs perform significantly better when the refinement of the output grid is high. This originates from the capacity of the PINN to impose a hard constraint based on the governing laws if a finer grid is available while maintaining consistency with the experimentally accessible data. Nonetheless, there are certain limitations. First, WS are fixed points in the domain of resolution, i.e. those points do not change in time and are typically sparsely distributed. The minimal theoretical spatial resolution can be estimated by the minimum distance between WS. Consequently, if there are events developing and disappearing in smaller distances, WS will not be able to track them. For the particular case analyzed here, the minimum spatial resolution corresponds to approximately 11.5 km. PINNs however have been proven to recover partially-lost information due to lack of spatial resolution when temporal resolution is at hand ^17, 22^. Hence, the readiness of the PINN to adapt to a rougher or finer spatial resolution than the original one can be analyzed. We set different spatial resolutions *R* = (0.05°,0.1°,0.2°) referred to latitude and longitude in the output grid, with approximate corresponding values of 5.5, 11 and 22 km, i.e. half of the given spatial resolution, the same order and its double. Figure 2 represents the different reconstructions for a typical instant given the different grid resolutions, showing great similarities among them while at the same time exposing the difficulties in recovering the slight fluctuations in the pressure field, which remains quasi-constant through a discrete time intake.

**Figure 3.**
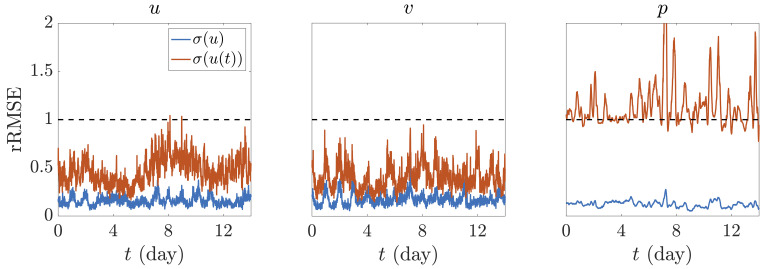
**rRMSE with respect to WS reference data normalized by** (blue) the standard deviation of the corresponding variable over the full timespan considered, *σ*( ) , and (red) the standard deviation over each specific time intake, *σ*( ( )).

To check the accuracy of the reconstruction, Figure 3 represents the PINN assimilation error (how accurately it learns the accessible reference data) per snapshot, i.e. per time intake, along the full timespan considered in the dataset (14 days). Here, the error is determined via the relative root mean squared error (rRMSE) of each variable, calculated as the square root of MSE in Equation 2 divided by the corresponding standard deviation, i.e. $\text{rRMSE}=\sqrt{\text{MSE}(\tilde{Z},Z)}/\sigma (Z)$ . Two different concepts of standard deviation have been imposed for the calculation of rRMSE: the total standard deviation of the complete dataset, i.e. for a period of 14 days, *σ*( ); and the standard deviation per intake, i.e. every 10 min, *σ*( ( )). It is expected that the PINN does a great job when assimilating information to a level of precision significantly below the overall standard deviation, whereas its accuracy should deteriorate when compared to the standard deviation for each particular time intake. This is the case of the two velocity components, which always maintain a good level of precision both below the discrete and overall standard deviation (represented by the horizontal dashed line at value rRMSE = 1). However, a major limitation occurs for the pressure assimilation. Indeed, as wind fluctuates more frequently, the standard deviation of the full dataset is on average very similar to that of one specific time snapshot. For pressure, on the contrary, its variation over a single discrete snapshot is minimal when compared to the overall fluctuation over 14 days. In fact, pressure changes in the timespan of 10 min are barely noticeable. Thus, the PINN is very accurate when measuring fluctuations over the full range of variation, but very limited when assimilating slight changes over a single discrete time.

The overall results for the full dataset may be checked in Table 1. Here, the rRMSE considers a period of 14 days and the 21 available WS. The error commited by the NS equations, calculated as $\sqrt{e_{1}^{2}+e_{2}^{2}+e_{3}^{2}}$ , is also indicated. As it can be noted from the aforementioned table, increasing the resolution of the output grid is beneficial since the PINN is able to properly embed the WS measurements in an easier manner. This is reflected by a decreasing rRMSE in every variable for a finer spatial resolution. However, a finer grid entails a higher uncertainty when calculating spatial derivatives, resulting in higher residual of the NS equations, which are subject to the inaccuracy of the reference measurements and the precision at which the PINN is able to assimilate them. However, we can conclude that, overall, increasing the spatial resolution of the output grid works in favor of a more accurate reconstruction. Indeed, when the selected resolution is of the same order of magnitude as the one of the reference data (or rougher), the PINN starts experiencing difficulties when assimilating WS reference information while enforcing in parallel the compliance with NS equations.

**Table 1.** Errors with respect to WS measured data and NS residuals over the reconstructed domain. The average value of all the errors for each spatial resolution is included for reference purposes.

*R*(°)	0.05	0.1	0.2
rRMSE( *u*)	0.1673	0.1724	0.1756
rRMSE( *v*)	0.1857	0.1904	0.1962
rRMSE( *p*)	0.1268	0.1280	0.1331
NS	0.1559	0.1530	0.1462
Average	0.1589	0.1609	0.1628

### 3.2. Tuning the strength of the physics constraint

The intensity at which the physics constraint is applied during the training of the PINN is a hyperparameter that has been demonstrated to have an insightful effect on the precision of the final reconstruction. In fact, recent studies show that this adaptability improves the performance of different NNs when reference data are very noisy or inaccurate ^15, 18, 33^. This hyperparameter, typically named *λ*, is incorporated into the loss function and indicates the rate of regularization that is desired on the final reconstruction. The corrected contribution to the total loss function ( Equation 3) may be written as ${ℒ}_{\text{NS}}^{\text{*}}$ = *λ* ℒ _NS_, resulting in an updated definition, which now reads ℒ = *ω*_NS_ ${ℒ}_{\text{NS}}^{\text{*}}$ + ℒ _WS_ = *ω*_NS_ *λ* ℒ _NS_ + ℒ _WS_. It is important to note that, in this definition, the contribution ℒ _NS_ is calculated using the same methodology as explained in Section 2 and that the weights ω_*_ need be updated to incorporate the effect of *λ*, i.e. ω_*_ = ℒ _*_/( *λ* ℒ _NS_ + ℒ *_u_* + ℒ *_v_* + ℒ *_p_*). Consequently, *λ* = 0 represents a pure data assimilation methodology, i.e. the PINN does not enforce any physics regularization during its training and the final outcome will only be accurate on the WS locations. Therefore, information at any other point in the field of reconstruction would not be necessarily reliable. Increasing values of *λ* indicate a stronger enforcement of the physics constraints, with *λ* = 1 corresponding to the standard definition of the loss function expressed in Equation 3. Two main tendencies may be approached with this hyperparameter: *λ* < 1 indicates that more importance is given to the assimilation of WS measurements and less to the regularization via physics constraints, whereas *λ* > 1 performs inversely, i.e. the PINN is directed to reconstruct a field that is more physically viable than a better match to the reference information. Note that *λ* shall not be set to negative values by definition. Nonetheless, any configuration of *λ* except its null value will incur in a certain regularization via physics constraints, which influences the way the PINN embeds the reference data since those values are prone to be affected by noise. These inaccuracies in the original dataset may reach up to levels of 40% in some instances ^34, 35^. The imposition of NS may contribute to the partial correction of those errors, as the physics constraints allow for a regularization of the original data ^22, 33, 36^. This regularization comes at the expense of never achieving a null loss value in Equation 3: a perfect assimilation of the reference data, i.e. ℒ _WS_ = 0 would never be compatible with ℒ _NS_ = 0, and viceversa. From laminar to slightly turbulent regimes, PINNs have been shown to adequately correct experimental errors ^22, 33^, achieving in all cases an excellent final reconstruction. Table 2, Table 3 and Table 4 collect the errors obtained during the training of several PINNs varying the different degrees of regularization via *λ* for different spatial resolution levels. Again, the errors with respect to the reference data are indicated via rRMSE and the error with respect to NS is indicated as the square root of the summation of the squared residuals for consistency, i.e. $\sqrt{e_{1}^{2}+e_{2}^{2}+e_{3}^{2}}$ .

**Table 2.** Errors with respect to WS data and NS over the full domain with spatial resolution *R* = 0.2° for different levels of *λ* regularization. In blue, the optimal configuration is highlighted.

*λ*	0	0.1	0.5	1	2	10
rRMSE( *u*)	0.1565	0.1677	0.1735	0.1756	0.1829	0.2526
rRMSE( *v*)	0.1721	0.1907	0.1935	0.1962	0.2017	0.2774
rRMSE( *p*)	0.1119	0.1261	0.1282	0.1331	0.1335	0.1716
NS	9.6788	0.4328	0.2029	0.1462	0.1099	0.0668
Average	2.5298	0.2293	0.1745	0.1628	0.1570	0.1921

**Table 3.** Errors with respect to WS data and NS over the full domain with spatial resolution *R* = 0.1° for different levels of *λ* regularization. In blue, the optimal configuration is highlighted.

*λ*	0	0.1	0.5	1	2	10
rRMSE( *u*)	0.1565	0.1639	0.1709	0.1724	0.1845	0.2204
rRMSE( *v*)	0.1721	0.1847	0.1899	0.1904	0.2033	0.2462
rRMSE( *p*)	0.1119	0.1253	0.1288	0.1280	0.1318	0.1530
NS	9.6788	0.4435	0.2134	0.1530	0.1159	0.0621
Average	2.5298	0.2294	0.1758	0.1609	0.1589	0.1704

**Table 4.** Errors with respect to WS data and NS over the full domain with spatial resolution *R* = 0.05° for different levels of *λ* regularization. In blue, the optimal configuration is highlighted.

*λ*	0	0.1	0.5	1	2	10
rRMSE( *u*)	0.1565	0.1605	0.1643	0.1673	0.1796	0.2216
rRMSE( *v*)	0.1721	0.1806	0.1826	0.1857	0.2005	0.2388
rRMSE( *p*)	0.1119	0.1204	0.1232	0.1268	0.1305	0.1514
NS	9.6788	0.4373	0.2151	0.1559	0.1190	0.0619
Average	2.5298	0.2247	0.1713	0.1589	0.1574	0.1684

**Figure 4.**
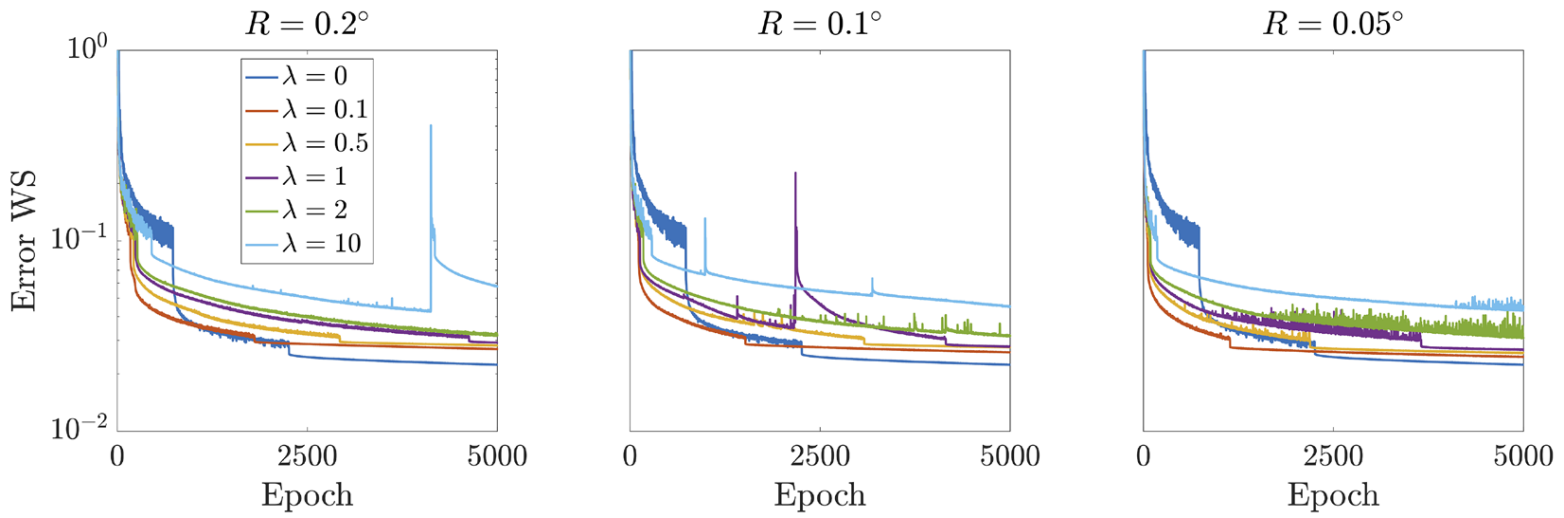
Error evolution regarding experimentally accessible data during PINN training for different spatial resolutions, computed as the average of each loss contribution with respect to the three reference variables, i.e. ( ℒ *_u_* + ℒ *_v_* + ℒ *_p_*)/3. Independently of the value of *λ*, all curves reach a plateau when an equilibrium has been achieved between the efforts put by the PINN to reduce the error with respect to the NS while assimilating scarce noisy data.

As a common trend for the different spatial resolutions, increasing values of *λ* reduce the error of the NS equations to the detriment of the rRMSE of the reference variables. However, when looking at the average error output it is worth noticing that the PINN significantly improves its performance when *λ* ≈ 2 for all levels of spatial resolution. This is an indicator that when reference data are noisy and scarce, more importance must be paid to the regularizing constraint instead of the assimilation of reference information. This is reflected in Figure 4, in which the loss contributions during the training of the PINN are disclosed for different levels of *λ*. At some point during training, the PINN reaches a plateau in which an equilibrium has been found between the two counteracting effects discussed before: the imposition of the physics constraints and the compliance with ‘noisy’ accessible data. This plateau shows a higher error with respect to the reference information for increasing values of *λ*. This originates from the fact that more importance is given to the regularization, and therefore, the PINN is more inclined to reduce the error with respect to the NS equations rather than assimilate the given data. In conclusion, if reference information is suspected of being inaccurate, more effort needs to be oriented to the embedding of the physics regularization, which may partially compensate for the original data errors. Nevertheless, it is important to note that this regularization factor needs not be too high, since for those cases, an excessive deviation with respect to the reference information may occur, impeding a final reconstruction in agreement with experimentally accessible data.

### 3.3. Validation of the weather model via PINN

As discussed in previous subsections, weather information is not readily available in locations where there are no recording stations. As a result, it is extremely difficult to reconstruct the weather flow and find useful information for comparison and validation purposes, since available data would be based on simulations, which already incorporate some hypothesis of the reconstructed behavior. Given such a complex scenario, the only plausible method for a strict validation of our methodology would be based on the elimination of weather stations from the training dataset and refer to them as test cases, i.e. estimate wind velocity and pressure at the location of those test stations, where actual measurements are readily available.

**Figure 5.**
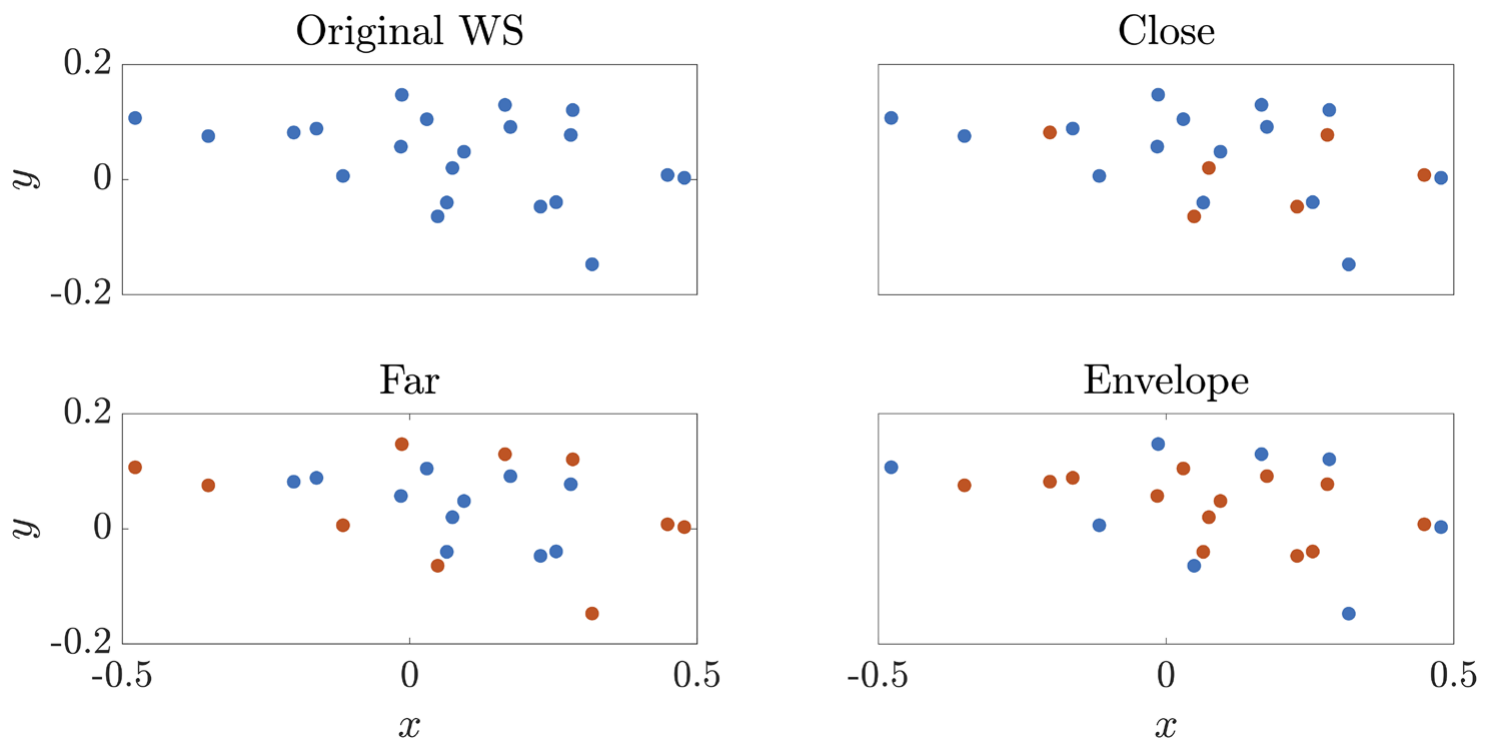
Description of selected WS for validation purposes for each test case. In blue, the retained WS for training are highlighted, whereas those in red are removed and only used for testing (and therefore are not included in the training dataset).

The proposed methodology will be tested on different cases to check its integrity when operating on weather data that is completely in or out of the field of measurement. For that purpose, three test scenarios are established based on the removal of WS very close to one another, very far from one another and the ones contained within an envelope. A representation of each case scenario is shown in Figure 5 for clarity purposes, with blue dots indicating the retained WS for training and red dots the removed ones (and kept for testing).

The ‘close’ scenario is particularly interesting because it proves that the regularization is done correctly. Any physics constraint should be able to project a precise behavior in points close to the ones used as reference. This is the first benchmark that should be addressed to validate the reconstruction capacity of the PINN. In this case, one wants to demonstrate that the PINN is able to detect changes in the near field. For that purpose, the rRMSE corresponding to the test WS is calculated for each variable. Results are summarized in Table 5 and compared to other approximation methods, such as linear interpolation, nearest and natural neighbor, and cubic and spline interpolation.

**Table 5.** Errors on test WS for the ‘close’ scenario calculated as rRMSE for each flow variable. The NS error is represented by the square root of the summation of squared residuals from the NS equations, i.e. $\sqrt{e_{1}^{2}+e_{2}^{2}+e_{3}^{2}}$ . The comparison to other methodologies is also indicated.

Method	PINN( *R* = 0.05°)	PINN( *R* = 0.1°)	PINN( *R* = 0.2°)	Linear	Nearest	Natural	Cubic	Spline
rRMSE( *u*)	0.3936	0.3896	0.4321	0.3589	0.4688	0.3498	0.3908	0.4367
rRMSE( *v*)	0.5024	0.4821	0.5052	0.4851	0.5351	0.4797	0.5033	0.5104
rRMSE( *p*)	0.1125	0.1182	0.1145	0.0640	0.0791	0.0639	0.0631	0.0702
NS	0.1072	0.1068	0.1051	3.2114	5.0232	2.8980	3.2357	2.4120
Average	0.2789	0.2742	0.2892	1.0298	1.5266	0.9479	1.0482	0.8573

Here, PINNs are able to perform similarly to other interpolation methodologies. This is expected since the stations that have been removed are all in the vicinity of the ones used during training, so big changes in the velocity and pressure values are not anticipated. It is very important to notice that rRSMEs are calculated based on the original values measured by the WS, which may be inaccurate. This adds another layer of uncertainty to perform a proper comparison, since the PINN has been shown to partially compensate for experimental / measuring errors. As a result, the PINN is presumed to perform better than reflected here. A direct interpolation using the proposed alternative methodologies does not compensate for errors, and therefore their predictions are assumed to be as noisy as the reference data. This is one of the main advantages of using PINNs: noise and errors in the original database are partially taken care of ^22^, improving the overall performance. In addition, even though all the interpolators used here for comparison show a high predictability capacity, they are not able to incorporate any physics regularization, i.e. the error with respect to NS equations is orders of magnitude higher than that of the PINN. This is an added value when used in other scenarios in which the points of interest are not in the vicinity of the reference ones.

This is particularly the case of the ‘far’ scenario, in which the performance of the PINN for reconstructing values far away from the set of training WS is exposed. The results are summarized in Table 6. In this scenario, the PINN proves to be remarkably more accurate than any other interpolation method (note that spline and cubic interpolators are not designed to be used as extrapolators and, therefore, no error is calculated). Both the errors with respect to the test WS and the NS residuals are significantly reduced as compared to those from the alternative interpolators. This validation is of extreme importance because it demonstrates the viability of using PINNs as regularizers even when there is lack of spatial and time resolution: whereas standard computational fluid dynamics (CFD) may incur in a significant amount of computational cost and time, PINNs show a high precision with a remarkable reduction in the computational effort, being significantly better than any other interpolation methodology that does not account for physics constraints.

**Table 6.** Errors on test WS for the ‘far’ scenario. Labels follow the same description as in Table 5.

Method	PINN( *R* = 0.05°)	PINN( *R* = 0.1°)	PINN( *R* = 0.2°)	Linear	Nearest	Natural	Cubic	Spline
rRMSE( *u*)	0.5110	0.4461	0.4439	1.4387	0.4923	–	–	0.7071
rRMSE( *v*)	0.6273	0.6085	0.6812	1.4285	0.5315	–	–	0.9045
rRMSE( *p*)	0.1585	0.1632	0.1626	0.1061	0.0936	–	–	0.3364
NS	0.1069	0.1046	0.1021	5.9104	3.3765	2.8125	3.2303	4.9466
Average	0.3509	0.3306	0.3474	2.2209	1.1235	–	–	1.7237

**Figure 6.**
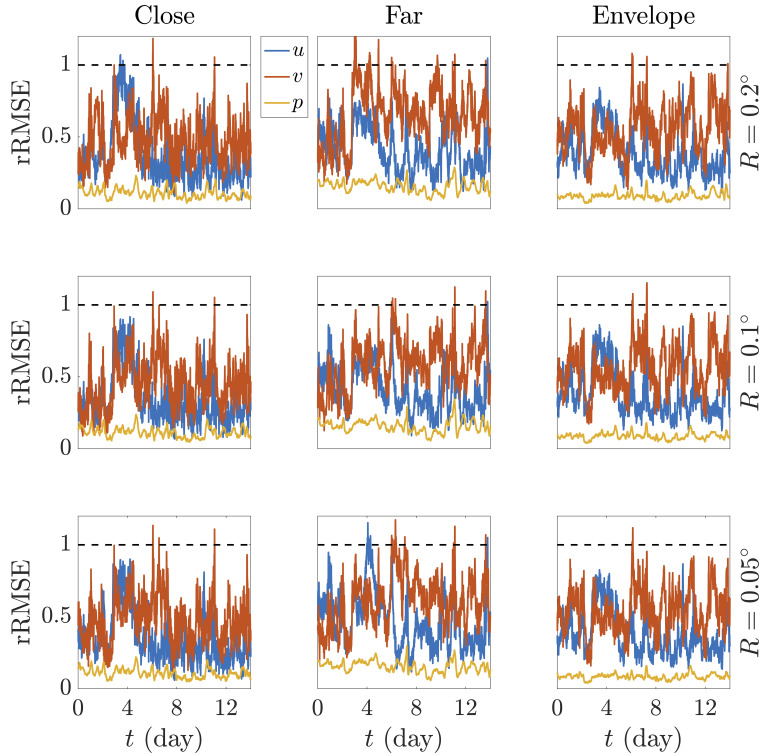
Evolution over the reconstructed time of the rRMSE of the predicted variables at the test WS for each validation case scenario, namely ‘close’, ‘far’ and ‘envelope’. Here, the standard deviation over the complete timespan, i.e. *σ*( ), has been used for dimensionality purposes when calculating rRMSE. The horizontal dotted black line indicates the threshold upon which estimated values are considered inaccurate.

Finally, the ability to infer information from a certain domain if a WS envelope is selected is assessed. In this last test, only WS located at the rim of the set of available WS are allowed during training, i.e. the selected WS form an envelope over the region of interest. A comparison between the use of PINN with different grid resolution and other methodologies is indicated in Table 7. PINNs show again an excellent resolution performance, indicating that the regularization via NS equations is properly embedded into the system. A high accuracy when predicting flow variables at the test WS is always achieved. Nevertheless, there are other simpler methodologies which provide similar forecasting capabilities. Again, we must note that these alternative interpolators do not disclose any physics behavior, and therefore, the error with respect to the NS is at least one order of magnitude higher than that provided by the PINN. Therefore, we demonstrate the higher efficiency of the PINN when compared to other standard interpolation techniques. One may discuss that the major improvement from the use of the PINN originates from the noise compensation / cancellation, as already discussed in 22. Figure 6 shows the rRMSE over the reconstructed timespan for the test WS in each validation scenario. A reference horizontal line at value rRSME = 1 serves as a threshold to determine whether the outcome of the PINN may be considered accurate (below threshold) or not (over threshold).

**Table 7.** Errors on test WS for the ‘envelope’ scenario. Labels follow the same description as in Table 5.

Method	PINN( *R* = 0.05°)	PINN( *R* = 0.1°)	PINN( *R* = 0.2°)	Linear	Nearest	Natural	Cubic	Spline
rRMSE( *u*)	0.4096	0.4166	0.4116	0.4154	0.4972	0.4070	0.4300	0.4359
rRMSE( *v*)	0.5636	0.5795	0.5774	0.5543	0.6863	0.5357	0.5663	0.5563
rRMSE( *p*)	0.0868	0.0908	0.0943	0.0543	0.1102	0.0526	0.0591	0.0661
NS	0.1058	0.1077	0.1122	2.9356	6.3034	2.7086	2.7800	2.4245
Average	0.2914	0.2987	0.2989	0.9899	1.8993	0.9260	0.9588	0.8707

Note that wind velocity is more challenging to correctly predict as compared to pressure. As already discussed, this is due to the fact that pressure is usually a very stable variable which barely fluctuates within a short timespan. This allows the PINN to accommodate the reference data in a more effective manner and therefore show a great ability to estimate pressure values at other stations, which would not significantly differ. That stability does not occur with wind, which oscillates more frequently. Once more, note that errors are measured with respect to the real values given by WS, which are intrinsically affected by noise. If perfectly accurate values with no noise were readily at hand, the error curves would be presumably improved. Nonetheless, the capacity of PINNs to embed physics constraints still exhibits its main advantage when compared to other methodologies that, even precise enough for a fast estimation of low-fluctuating phenomena, do not pose any regularization on the original data. PINNs show an exceptional reconstruction accuracy both when interpolating and extrapolating information in the field of measurement thanks to their ability to calculate precise derivatives and consequently impose a physics background.

## 4. Conclusions

In this article, we have discussed the potentiality of using PINNs for the accurate reconstruction of wind and pressure fields from WS recordings. Different parameters have been incorporated so as to gain a deep insight on the way a PINN learns relevant data and applies a regularization based on several physics constraints.

First of all, the scarcity of accurate information poses a limitation on the capacity of the PINN to both assimilate the experimentally accessible data while at the same time enforcing the compliance with physics laws. This impediment originates from the data sources, based on WS that are fixed in space and provide recordings every several minutes. Therefore, the first drawback that is encountered is the limited time and spatial resolutions. Assumptions can be made to estimate if the available time resolution is fine enough to capture relevant phenomena, which is the case for the majority of events that are disclosed in this article. Concerning spatial resolution, the ratio of reference data to the final output grid is an important parameter. For 14 days of recording with a time interval of 10 min and 21 available WS, this ratio corresponds to 1.20, 4.25 and 13.13% for an output resolution of *R* = 0.05°, 0.1° and 0.2°, respectively. This small percentage is extremely relevant and plays a significant role in the ability of the PINN to properly assimilate the reference values and incorporate them into the learning process when constraints are imposed in the rest of the domain. The strength at which the physics constraint is imposed plays also a remarkably relevant role. This is achieved by the hypertuning of the adjustable parameter *λ*. Values smaller than the unity indicate stronger efforts to reconstruct a field which more accurately resembles the reference information, whereas higher values expose a stronger regularization via physics. For cases in which there is scarcity of data, which may not necessarily be accurate, *λ* is recommended to be always slightly higher than the unity, though not that high that the output field may totally differ from the accessible information. The higher the scarcity or the inaccuracy of the original reference values, the higher *λ* should be, allowing the PINN to partially compensate for given errors.

Finally, PINNs are able to adequately perform when reconstructing the velocity and pressure domain as compared to other interpolators. Two effects play in our advantage: the reduction of noise and inaccuracies from the original dataset, and the capacity of the PINN to extrapolate information with precision due to the regularization by enforcement of physics constraints. Whereas the PINN achieves relatively similar results than other interpolators when estimating values very close to the location of the WS or within an envelope, they outperform other methodologies when predicting the behavior of the atmosphere far away from the cluster formed by the source WS. The enhancement of data predictability is therefore justified and the regularization provided by the NS equations suffices for a correct weather estimation in other regions not covered by the WS. Other interpolators fail at this task, since the capability of extracting precise information at other locations is highly dependent on the precision of the reference points, failing drastically at extrapolating outside the region of training.

PINNs have also been shown to be a very powerful tool when estimating information at other locations and smoothing fluctuations that may occur in the fluid field. This works in our advantage when calculating temporal and spatial derivatives, essential to comply with the physics constraints imposed by the NS equations. However, a major limitation originates from this smoothing effect: rapid events developing and disappearing below the time resolution of the original dataset and local events occurring in a region below the spatial resolution (and not captured by a WS) would not be reconstructed nor recovered. PINNs are thus useful neural networks to partially compensate for source errors, to accurately estimate flow properties in other areas in and outside the field of view, and to recover average field behavior. Consequently, PINNs have been proven to be a useful asset when used for reconstructing a complete physically - based wind and pressure field from scarce WS data with precision. Nevertheless, they are still very dependent on the quality of the reference information. Sparsity and inaccuracy of data is certainly the case in most real situations. This first attempt to fully reconstruct a weather field with the use of a PINN shows promising results to improve meteorological predictions in a rapid manner. Therefore, it opens many doors for future research regarding the application of physics constraints to embed and regularize weather fields.

## Ethics and consent

Ethical approval and consent were not required.

## Data Availability

Information used in this article has been extracted from a dataset acquired by the Universidad Carlos III de Madrid under the scope of project ALARM. The original dataset was directly purchased from the Royal Meteorological Institute of Belgium and is therefore of private use and may not be shared without the explicit permission of the provider. As a courtesy, the 14-day segment analyzed here and the corresponding script to train the PINN can be found at the dedicated repositories in Zenodo and GitHub, respectively. The meteorological variables are provided by the Royal Meteorological Institute of Belgium for the vicinity of the Brussels-Zaventem airport during 2018. Temporal resolution of the data is 10 minutes. Relevant information includes the geolocation of each weather station, the measured wind speed and direction, pressure, temperature and the associated time of the recording. Zenodo: Dataset of 'Physics-informed neural networks for high-resolution weather reconstruction from sparse weather stations'. https://doi.org/10.5281/zenodo.10723261 ^37^. Data are available under the terms of the Creative Commons Attribution 4.0 International license (CC-BY 4.0)

## Software availability

Scripts have been coded via Python and Tensorflow 2, and are publicly available through Zenodo and GitHub repositories.

Zenodo: Scripts and video of 'Physics-informed neural networks for high-resolution weather reconstruction from sparse weather stations'. https://doi.org/10.5281/zenodo.10911856 ^38^.

GitHub: PINNs-for-high-resolution-weather-reconstruction-from-sparse-weather-stations.

https://github.com/AlvaroMS90/PINNs-for-high-resolution-weather-reconstruction-from-sparse-weather-stations

Scripts are available under the terms of the Creative Commons Attribution 4.0 International license (CC-BY 4.0).
