# Supplementary material for: Physics-informed neural networks for high-resolution weather reconstruction from sparse weather stations
Source: Open Res Eur. 2024 May 10;4:99. [Version 1] doi: 10.12688/openreseurope.17388.1 (PMC11308984; doi:10.12688/openreseurope.17388.1)
Supplement: Peer review history

# Reviewer report: Reviewer response for version 1

**DOI:** 10.21956/openreseurope.18793.r41852

**Published:** 2024-08-05

**Authors:** Shashank Subramanian

**Affiliations:** 1 Lawrence Berkeley National Laboratory, Berkeley, USA

The paper introduces PINNs to reconstruct a 2D atmospheric state (velocities and pressure) at low altitudes using sparse supervised data from weather stations and self-supervision from the Navier-Stokes PDE. The authors apply the PINN on a dataset of 14 days observations in a small area (Brussels) and investigate several aspects of its performance: accuracy w.r.t spatial resolution, importance of the physics regularization, and effect of the location of weather stations chosen for the test dataset. The paper is clear and overall an interesting paper that focuses on an important application of PINNs.

 Comments and questions:

 1. Some of the experimental settings could be explained more. Example: what is the train/validation/test split for the first two settings, are the full 14 days used for training, what are the IC/BCs assumed for PINN training?

 2. 14 days in a dataset seem very restrictive and a concern would be overfitting to this dataset. It would be good to see the results with training on a larger time-scale (years) and testing on newer years for these weather stations. It is difficult to judge the PINN without either other regions or extended/different time-scales.

 3. Relating to 2, stronger baselines would help strengthen the paper: ERA5 reconstructions, physics-solver reconstructions (NS solver or any other SOTA simulation which can be data assimilated). While these two have high computational cost, other baselines like trained weather surrogates (GraphCast, FourCastNet, Pangu etc) can also be compared since the models are open-sourced and they are very cheap to apply. 

 4. To motivate PINNs, there could be a discussion on how they compare to the above surrogates and/or downscaling them with diffusion models (or other generative models) to get even finer features. The latter are especially motivated for capturing extreme phenomena which according to the authors would be smoothed out in the PINN approach.

 5. For the R experiment, it would be interesting to see how the trained PINN performs on different resolutions in inference. Further, I believe the authors assume a uniform grid of collocation points. It would be interesting to see random samples (that could be adapted based on the PDE residual) for these points during training and how they change the result (if at all)

 6. In all tables, how do I interpret an NS residual of 0.15 etc? Is it small? It might be good to show a simulated result as well in the figures as well as the PDE residual map at the end of training to see where the residual is high.

 7. Very minor: the \lambda tables might be easier to read if they were a figure to see the tradeoffs.

Is the study design appropriate and does the work have academic merit?

Partly

Is the work clearly and accurately presented and does it cite the current literature?

Partly

If applicable, is the statistical analysis and its interpretation appropriate?

Partly

Are all the source data underlying the results available to ensure full reproducibility?

Yes

Are the conclusions drawn adequately supported by the results?

Partly

Are sufficient details of methods and analysis provided to allow replication by others?

Partly

Reviewer Expertise:

Deep learning for science, HPC

I confirm that I have read this submission and believe that I have an appropriate level of expertise to confirm that it is of an acceptable scientific standard, however I have significant reservations, as outlined above.

# Reviewer report: Reviewer response for version 1

**DOI:** 10.21956/openreseurope.18793.r41854

**Published:** 2024-07-27

**Authors:** Mariana Clare

**Affiliations:** 1 European Centre for Medium-Range Weather Forecasts, Bonn, Germany

This article presents an interesting approach to data assimilation using Physically-informed Neural Networks, for a specific location in Belgium.

 Major

  

1. 14 days is not enough to evaluate a weather forecasting model. Generally models are evaluated against a whole season. Moreover it is not clear to me how you have split the training and test datasets in section 3.1 and 3.2
2. Although some basic interpolation methods are shown, there is no benchmark of how the PINN method compares against a standard data assimilation method. I think this is necessary in order to evaluate whether the PINN is a realistic alternative for other methods.
3. I think it would be good to see more spatial fields (like Figure 2) so that the reader can more easily compare the differences between the PINN and the basic interpolation methods which the authors claim are unphysical. Is this unphysicalness visible in the spatial field?
4. It would be good to test this method in a different location to see if it generalises, although I accept this may be difficult due to access to data.

Minor

  

1. I am a little bit confused by your emphasis on the accurate provision of weather information being useful for air traffic management, in the abstract. As you rightly point out in the introduction there are far more important reasons for accurate weather prediction such as mitigating impacts of extreme events.
2. The introduction is missing all the developments since 2022 of large scale data-driven weather forecasting models such as Pangu, GraphCast, FourCastNet and AIFS. Furthermore there have been studies on the physicalness of these models for example Hakim et al. (2024) and Bonavita (2024), which I think need to be mentioned. There has also been work using neural networks with data assimilation (see for example Melinc and Zaplotnik (2024)).
3. I think you could directly write the simplified version of the navier stokes equations, eliminating the vertical components (like you have in Figure 1). For this paper what is relevant is what the PINN is using and you can just add a reference for the Navier Stokes equations.
4. In Figure 3, what resolution are you using for the results?
5. What are the increased costs associated with increasing resolution? Does it scale linearly?
6. Figure 6: How have you determined that rRMSE=1 is a good accuracy threshold?

Is the study design appropriate and does the work have academic merit?

Yes

Is the work clearly and accurately presented and does it cite the current literature?

Partly

If applicable, is the statistical analysis and its interpretation appropriate?

Partly

Are all the source data underlying the results available to ensure full reproducibility?

Yes

Are the conclusions drawn adequately supported by the results?

Partly

Are sufficient details of methods and analysis provided to allow replication by others?

Yes

Reviewer Expertise:

Data-driven weather forecasting

I confirm that I have read this submission and believe that I have an appropriate level of expertise to confirm that it is of an acceptable scientific standard, however I have significant reservations, as outlined above.

# Reviewer report: Reviewer response for version 1

**DOI:** 10.21956/openreseurope.18793.r41858

**Published:** 2024-07-23

**Authors:** Hao Chen

**Affiliations:** 1 Beihang University, Beijing, Beijing, China

This manuscript proposed a PINN-based method for weather reconstruction from sparse weather station observations. The reviewer believes that the task studied in the manuscript is very interesting and has strong practical significance. The author has conducted a comprehensive review of the work related to PINN, and the logical flow in the introduction section is relatively clear. However, there are still some issues that need to be addressed. The specific suggestions are as follows:

 - As far as the reviewer knows, the basic motion of the atmosphere is described by the set of atmospheric motion equations. However, the differential equation constraints used in the manuscript only include the idealized NS (Navier-Stokes) equations. How can the effectiveness of these differential equation constraints be ensured in real-world scenarios under such conditions?

 - Directly obtaining meteorological field data from weather station observations is an interesting task. However, for general analysis fields or reanalysis fields, various observations, including satellite remote sensing and model outputs, are usually assimilated. How can these data be integrated within the current framework?

 - In the experimental section, the author compared the model performance under different hyperparameter settings but lacked a comparison with some publicly available meteorological analysis fields, such as ERA5. Please explain the reason for this.

 - At such a high resolution on the kilometer scale, terrain has a significant impact on meteorological variables. Have terrain-related factors been considered?

 - In addition to work related to PINNs, recent studies that combine meteorology with PINNs and focus on site observations should be mentioned and reviewed in the manuscript.

  [1]https://arxiv.org/abs/2401.04125

  [2]https://www.semanticscholar.org/paper/Deep-Learning-for-Day-Forecasts-from-Sparse-Andrychowicz-Espeholt/207c4b5a58d2b47a1e1b5f8a6877018a99f1bf68

Is the study design appropriate and does the work have academic merit?

Partly

Is the work clearly and accurately presented and does it cite the current literature?

Partly

If applicable, is the statistical analysis and its interpretation appropriate?

Not applicable

Are all the source data underlying the results available to ensure full reproducibility?

No

Are the conclusions drawn adequately supported by the results?

Partly

Are sufficient details of methods and analysis provided to allow replication by others?

Partly

Reviewer Expertise:

weather forecasting, data fusion, PINNs, remote sensing

I confirm that I have read this submission and believe that I have an appropriate level of expertise to state that I do not consider it to be of an acceptable scientific standard, for reasons outlined above.

# Reviewer report: Reviewer response for version 1

**DOI:** 10.21956/openreseurope.18793.r41094

**Published:** 2024-07-10

**Authors:** Subhamoy Chatterjee

**Affiliations:** 1 Southwest Research Institute, San Antonio, Texas, USA

This is an important work demonstrating the effectiveness of Physics Informed Neural Networks (PINN) in reconstructing high-resolution weather information using sparsely located weather stations. The paper provides insights into PINN performance by tuning grid resolution, changing the relative weightage of data assimilation vs. physics constraint in the loss function, and validating the model on unseen data. 

 Although minor I feel the paper will be benefited by addressing the following concerns-

 1) It is not explicitly mentioned in Section 3.3 what value of lambda was used for training the PINN in the three scenarios.

 2) It would be nice if the authors could add another test case where only a certain duration (e.g. 8-10 days) out of 14 days is included in the training set and the rest in the test set.

 3) The authors highlight that PINN plays a critical role in "the reduction of noise and inaccuracies from the original dataset" by regularization through physics constraints. To make this more explicit, it would be nice if the authors could create synthetic data following Navier-Stokes equations, add noise, and then train PINN on the noisy data to demonstrate the retrieval of the original information (not affected by noise) in comparison to the baseline methods.

 The paper is already in great shape and should be ready to be accepted for indexing after the above concerns are addressed.

Is the study design appropriate and does the work have academic merit?

Yes

Is the work clearly and accurately presented and does it cite the current literature?

Yes

If applicable, is the statistical analysis and its interpretation appropriate?

Yes

Are all the source data underlying the results available to ensure full reproducibility?

Yes

Are the conclusions drawn adequately supported by the results?

Yes

Are sufficient details of methods and analysis provided to allow replication by others?

Yes

Reviewer Expertise:

I specialize in novel applications of Machine Learning techniques on heliophysics problems. My current research problems range from inter-instrument data calibration, space weather forecasting, detection of solar transients, and interpretable generation of solar data.

I confirm that I have read this submission and believe that I have an appropriate level of expertise to confirm that it is of an acceptable scientific standard.
